# Supplementary material for: Structure of Myosin VI/Tom1 complex reveals a cargo recognition mode of Myosin VI for tethering
Source: Nat Commun. 2019 Aug 1;10:3459. doi: 10.1038/s41467-019-11481-6 (PMC6673701; doi:10.1038/s41467-019-11481-6)
Supplement: Supplementary file 1 — Supplementary Information [file 41467_2019_11481_MOESM1_ESM.pdf]

Structure of Myosin VI/Tom1 complex reveals a cargo recognition mode of Myosin VI for tethering

Hu et al.

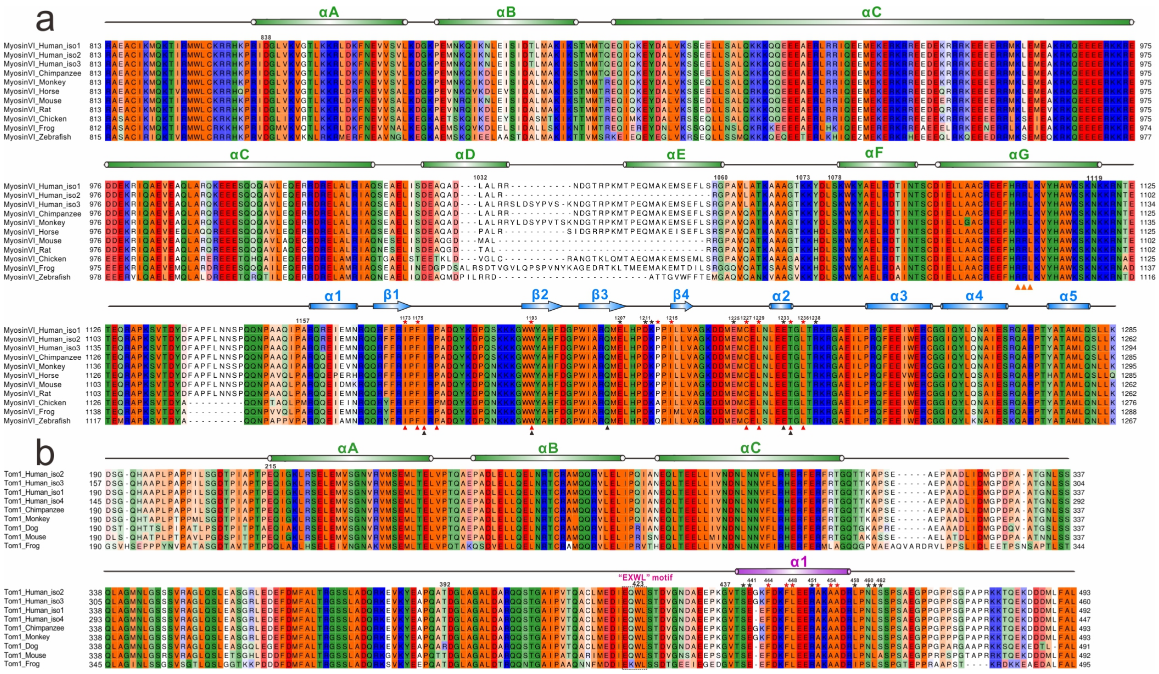

Supplementary Figure 1. Sequence alignment analyses of the Myosin VI tail region and the C-terminal part of Tom1. (**a**) Structure-based sequence alignment of Myosin VI tail region from different species. In this alignment, the conserved residues are highlighted by colors using software Jalview2.8.1 (http://www.jalview.org/). The interface residues that are crucial for the interaction with Tom1, are highlighted with black (polar interactions) or red (hydrophobic interactions) stars, while the residues that are involved in the interaction with the αA segment of Dab2 are labeled with black (polar interactions) or red (hydrophobic interactions) triangles. (**b**) Structure-based sequence alignment of Tom1 from different species. In this alignment, the conserved residues are highlighted by colors using software Jalview2.8.1 (http://www.jalview.org/). The interface residues of Tom1 that are crucial for the interaction with Myosin VI are highlighted with black (polar interactions) or red (hydrophobic interactions) stars. The mentioned EXWL motif in the main text is also indicated and highlighted with a black box.

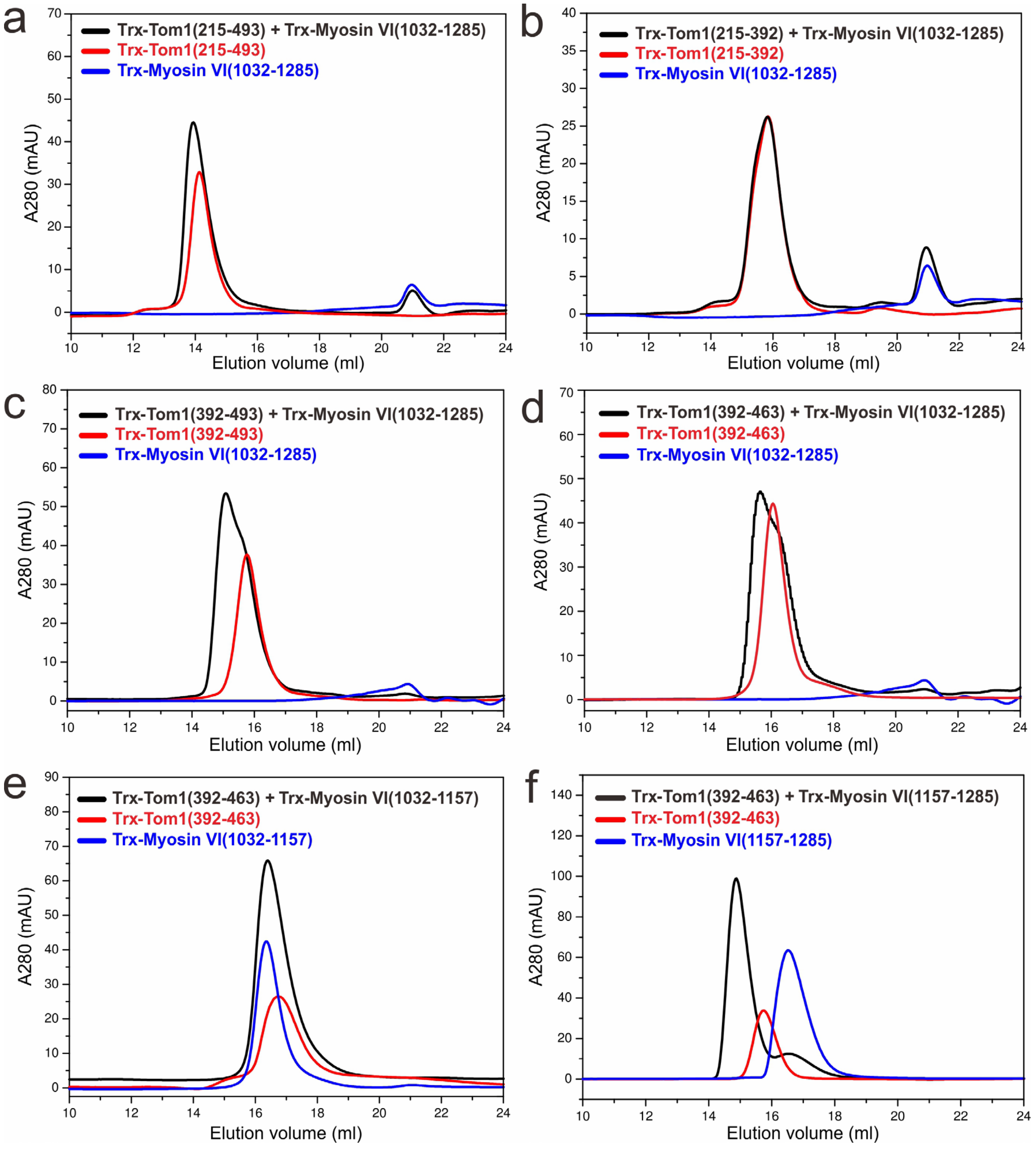

Supplementary Figure 2. Biochemical mappings of the interaction regions of Myosin VI and Tom1. (**a**-**d**) Analytical gel filtration chromatography analyses of the interactions between Myosin VI(1032-1285) and four C-terminal fragments of Tom1, Tom1(215-493) (**a**), Tom1(215-392) (**b**), Tom1(392-493) (**c**), and Tom1(392-463) (**d**). (**e** and **f**) Analytical gel filtration chromatography analyses of the interactions between Tom1(392-463) and

two Myosin VI fragments, Myosin VI(1032-1157) (the RRL motif of Myosin VI) (**e**), and Myosin VI(1157-1285) (the C-terminal CBD of Myosin VI) (**f**). Source data are provided as a Source Data file.

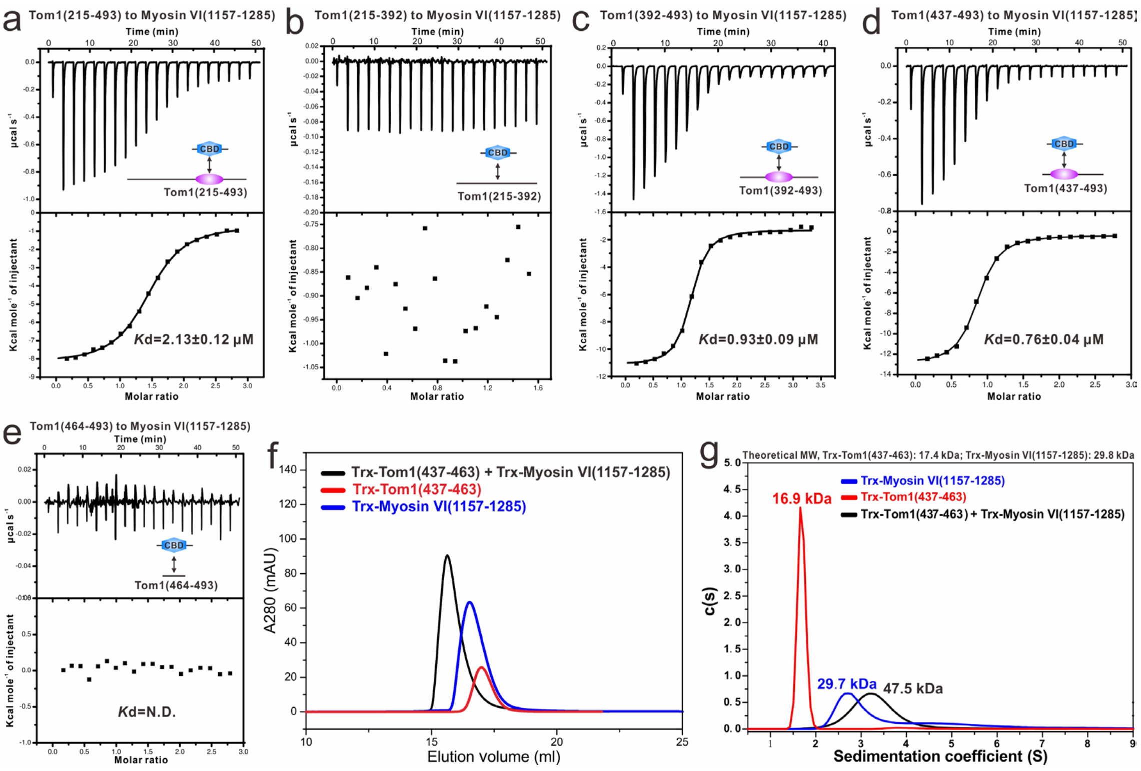

Supplementary Figure 3. Biochemical characterizations of the interactions of the C-terminal CBD of Myosin VI with different Tom1 fragments. (**a**-**e**) ITC-based measurements of the binding affinities of the C-terminal CBD of Myosin VI with Tom1(215-493) (**a**), Tom1(215-392) (**b**), Tom1(392-493) (**c**), Tom1(437-493) (**d**), Tom1(464-493) (**e**). *K*d values are the fitted dissociation constants with standard errors, when using the one-site binding model to fit the ITC data. Source data are provided as a Source Data file. (**f**) Analytical gel filtration chromatography analysis of the interaction between the C-terminal CBD of Myosin VI and Tom1 MBM. Source data are provided as a Source Data file. (**g**) Overlay plots of the sedimentation velocity data of the C-terminal

CBD of Myosin VI (blue), Tom1 MBM (red), and the C-terminal CBD of Myosin VI/Tom1 MBM complex (black). Source data are provided as a Source Data file. The results demonstrate that the monomeric C-terminal CBD of Myosin VI and Tom1 MBM can interact with each other to form a 1:1 stoichiometric complex in solution.

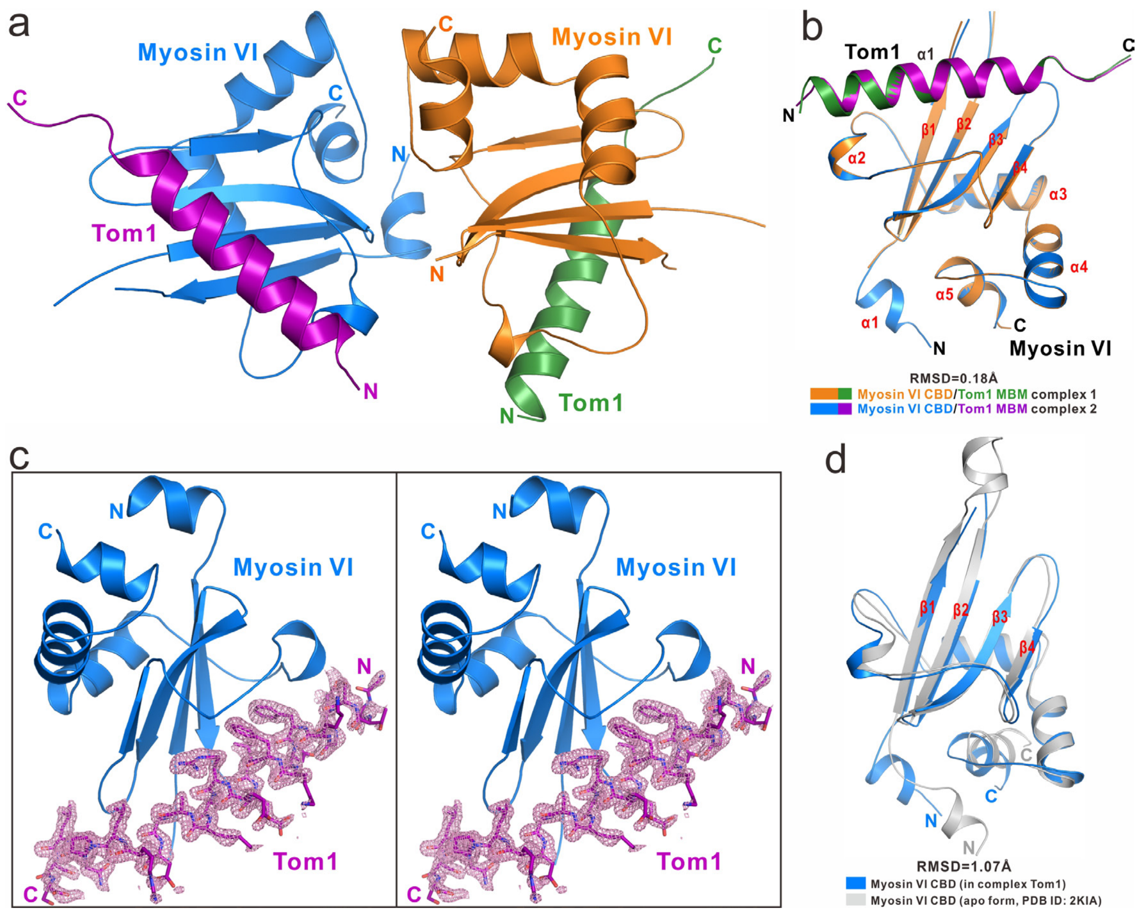

Supplementary Figure 4. Structural analyses of the C-terminal CBD of Myosin VI and Tom1 MBM complex. (**a**) Ribbon diagram showing the overall structures as well as the steric arrangements of two C-terminal CBD of Myosin VI and Tom1 MBM complexes in an asymmetric unit. (**b**) The comparison of the overall structures of two Myosin VI/Tom1 complexes in an asymmetric unit. (**c**) The F_O-F_C map of the Tom1 MBM region in the final structure of the Myosin VI/Tom1 complex showing that the

densities of Tom1 MBM can be clearly assigned. The electron density map is calculated by omitting the Tom1 MBM from the final PDB file and contoured at 2.0σ. The C-terminal CBD of Myosin VI molecule is shown in the cartoon mode, while the Tom1 MBM is shown in the stick mode. (**d**) Structural comparison of the C-terminal CBD of Myosin VI in the Myosin VI/Tom1 complex (blue) and its apo-form (grey, PDB ID: 2KIA)

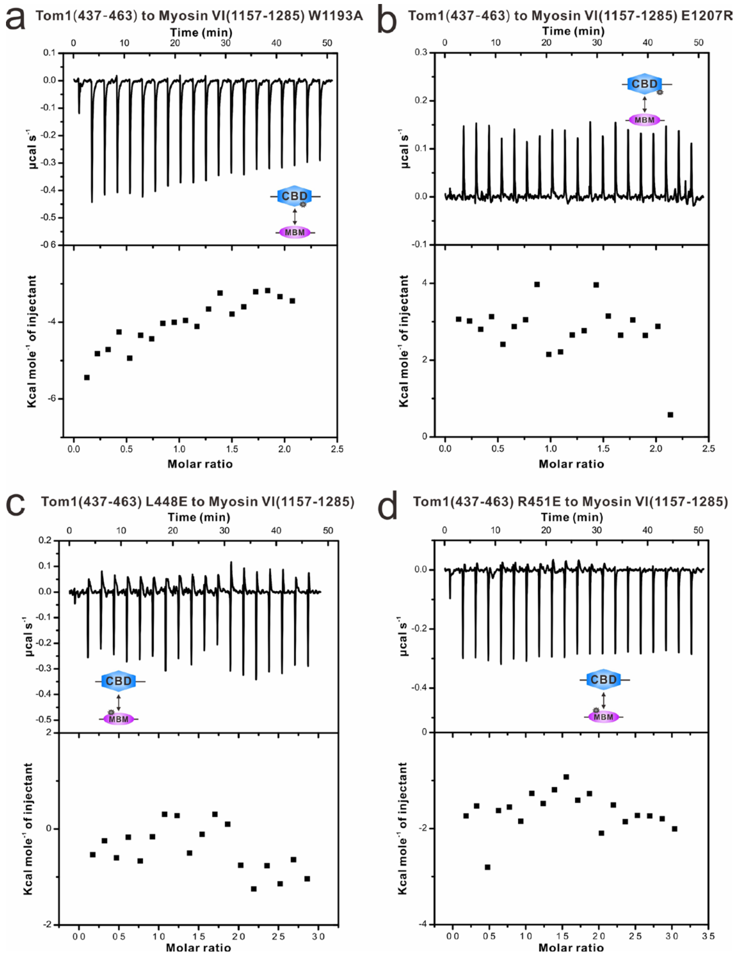

Supplementary Figure 5. Validations of the C-terminal CBD of Myosin VI and Tom1 MBM complex structure by mutations of key interface residues. (**a** and **b**) ITC-based measurements of the binding affinities of Tom1 MBM with the C-terminal

CBD of Myosin VI W1193A mutant (**a**), and E1207R mutant (**b**). (**c** and **d**) ITC-based measurements of the binding affinities of the C-terminal CBD of Myosin VI with Tom1 MBM L448E mutant (**c**), and R451E mutant (**d**). All these data confirm the specific interaction between the C-terminal CBD of Myosin VI and Tom1 MBM observed in the complex structure. Source data are provided as a Source Data file.

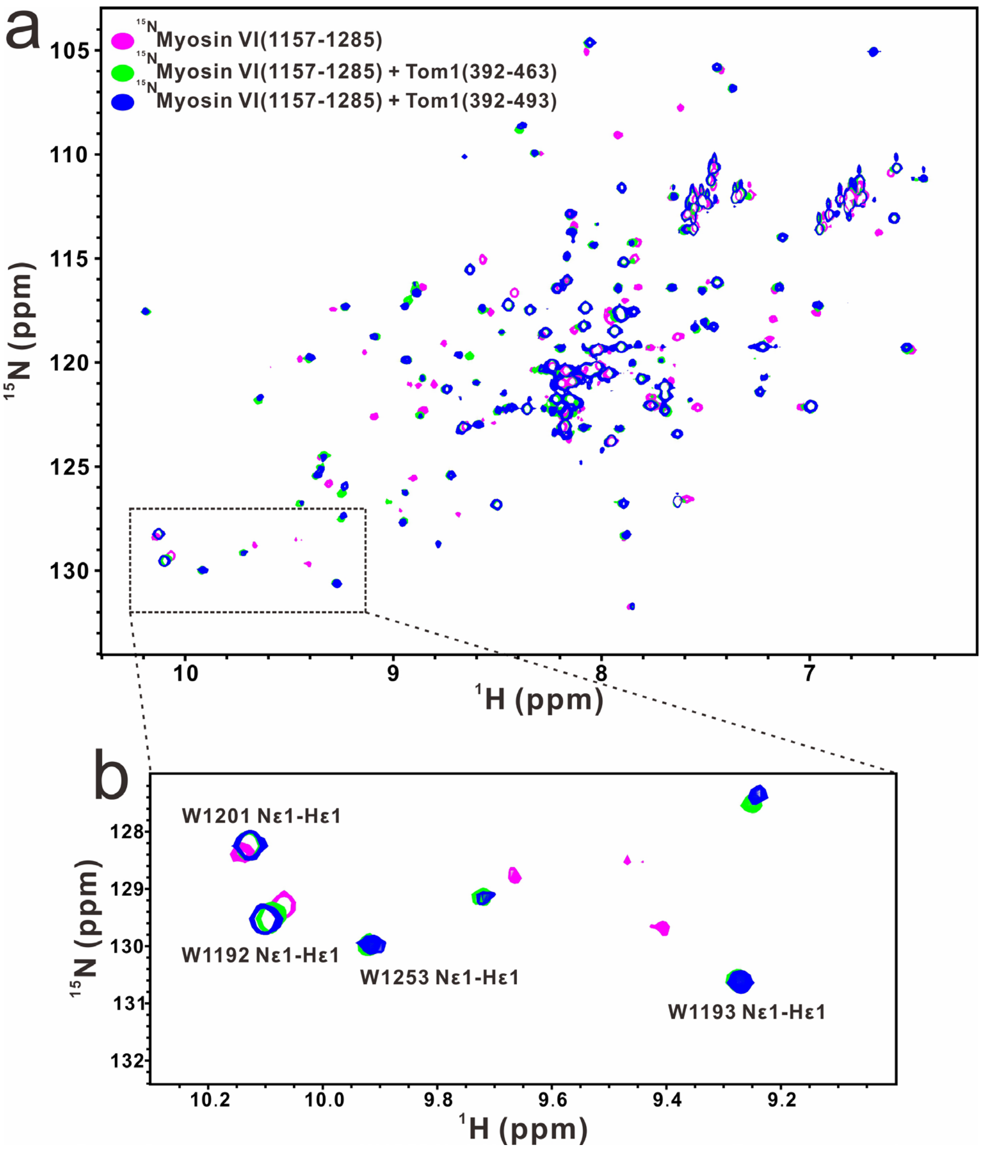

Supplementary Figure 6. The C-terminal extended region adjacent to the MBM of Tom1 is not involved in the interaction of Tom1 with the C-terminal CBD of Myosin

VI. (**a**) Superposition plots of the ^{1}H-^{15}N HSQC spectra of the C-terminal CBD of Myosin VI (magenta) and the domain saturated with Tom1(392-463) (green), or Tom1(392-493) (blue), showing that the inclusion of the C-terminal extended region adjacent to the MBM of Tom1 doesn't induce additionally significant chemical shift changes to the C-terminal CBD of Myosin VI. (**b**) The enlarged view of a selected region of the overlaid ^{1}H-^{15}N HSQC spectra in panel **a** containing several characteristic peaks corresponding to the side chains of W1192, W1193, W1201 and W1253 residues of the C-terminal CBD of Myosin VI. Notably, the W1192 residue is located at the site II of the C-terminal CBD of Myosin VI, while the W1193 residue is located at the site I of the C-terminal CBD of Myosin VI.

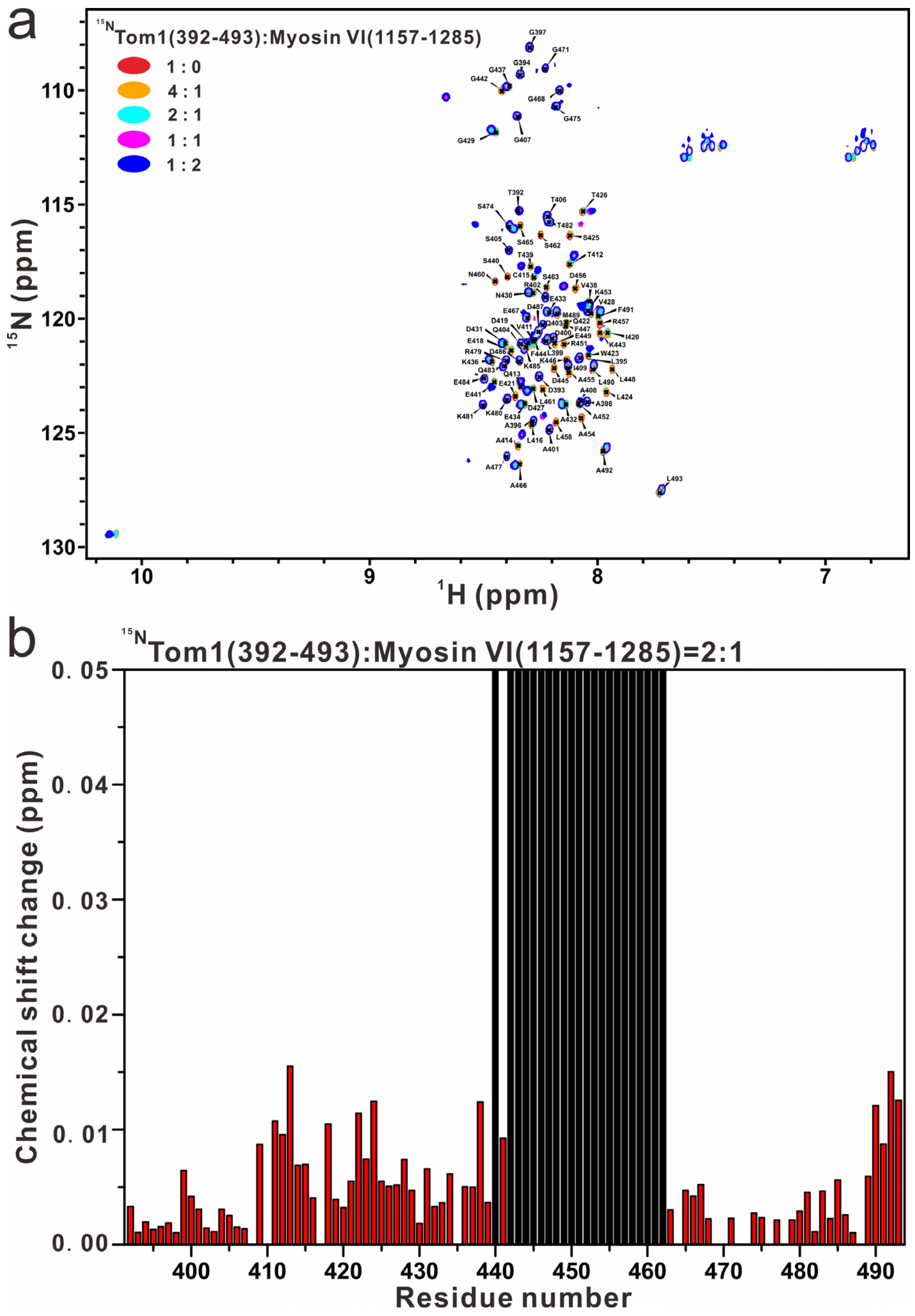

Supplementary Figure 7. NMR-based characterizations of the interaction between Tom1(392-493) and Myosin VI(1157-1285). (**a**) Superposition plots of the ^{1}H-^{15}N HSQC spectra of ^{15}N-labelled Tom1(392-493) titrated with the increasing molar ratios of un-labelled Myosin VI(1157-1285) proteins. (**b**) A plot of backbone amide chemical shift differences and peak-broadenings as a function of the residue number of Tom1(392-493)

between the wild-type and the protein titrated with Myosin VI(1157-1285) at the molar ratio of 2:1. In this representation, the residues with disappeared NMR peaks due to peak-broadenings are shown in black, and the combined ^{1}H and ^{15}N chemical shift changes are defined as:

$$(1)\ \Delta_{ppm} = [(\Delta\delta_{HN})^2 + (\Delta\delta_N \times \alpha_N)^2]^{1/2}$$

Where $\Delta\delta_{HN}$ and $\Delta\delta_N$ represent chemical shift differences of amide proton and nitrogen chemical shifts of the each residue of Tom1(392-493). The scaling factor (α_N) used to normalize the ^{1}H and ^{15}N chemical shift is 0.17. Source data are provided as a Source Data file.

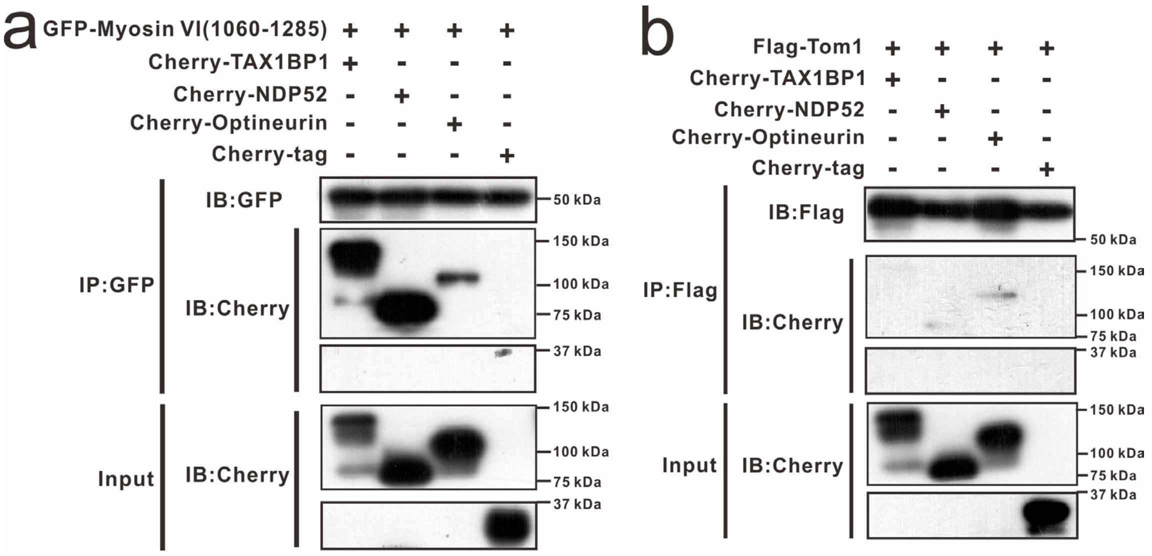

Supplementary Figure 8. Co-immunoprecipitation assays to characterize the interactions of Myosin VI and Tom1 with autophagy receptors, TAX1BP1, NDP52, and Optineurin. (**a**) A co-immunoprecipitation assay showing that Myosin VI(1060-1285) can specifically interact with autophagy receptors, TAX1BP1, NDP52 and Optineurin. (**b**) A co-immunoprecipitation assay showing that Tom1 is unable to interact

with autophagy receptors, TAX1BP1, NDP52, and Optineurin. In this assay, cell extracts were prepared from HEK293T cells co-transfected with different combinations of plasmids as indicated, and 5% of each extracts were used as loading controls (bottom panel). Source data are provided as a Source Data file.

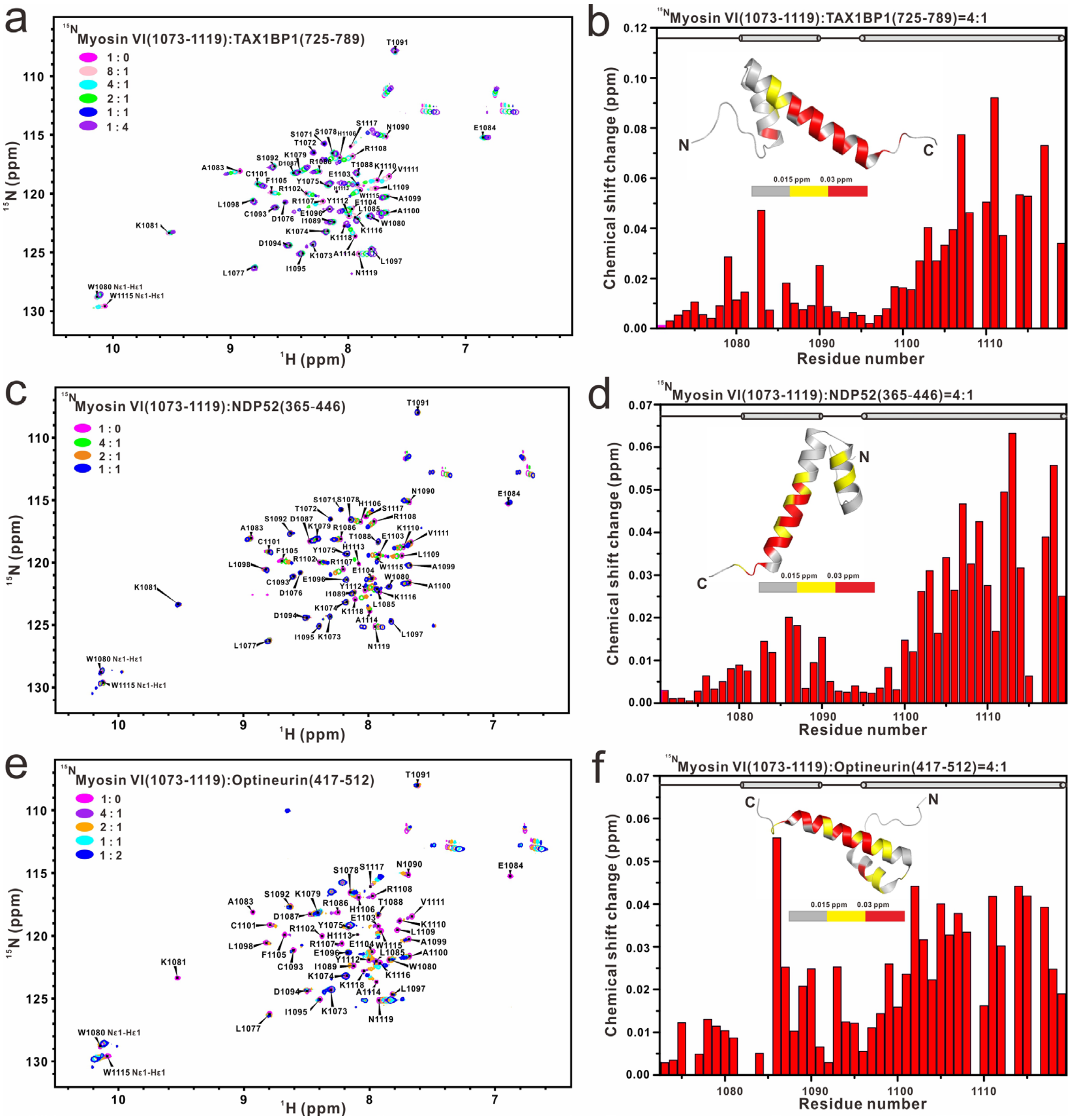

Supplementary Figure 9. NMR-based characterizations of the interactions between Myosin VI(1073-1119) and the autophagy receptors TAX1BP1, NDP52 and

Optineurin. (**a**, **c**, **e**) Superposition plots of the ^{1}H-^{15}N HSQC spectra of Myosin VI(1073-1119) titrated with TAX1BP1(725-789) (**a**), NDP52(365-446) (**c**), Optineurin(417-512) (**e**) at different molar ratios. (**b**, **d**, **f**) Plots of backbone amide chemical shift differences as a function of the residue number of Myosin VI(1073-1119) between the wild-type and the protein titrated with TAX1BP1(725-789) (**b**), NDP52(365-446) (**d**), Optineurin(417-512) (**f**) at the molar ratio of 4:1. In each panel, the secondary structures and amino acid sequence of Myosin VI(1073-1119) are also indicated at the top of the figure, and the insert shows the shift changes mapped onto a representative NMR structure of Myosin VI(1071-1122) (PDB ID: 2N10). In these representations, the combined ^{1}H and ^{15}N chemical shift changes are defined as:

$$(2)\ \Delta_{ppm} = [(\Delta\delta_{HN})^2 + (\Delta\delta_N \times \alpha_N)^2]^{1/2}$$

Where $\Delta\delta_{HN}$ and $\Delta\delta_N$ represent chemical shift differences of amide proton and nitrogen chemical shifts of the each residue of Myosin VI(1073-1119). The scaling factor (α_N) used to normalize the ^{1}H and ^{15}N chemical shift is 0.17. Source data are provided as a Source Data file.

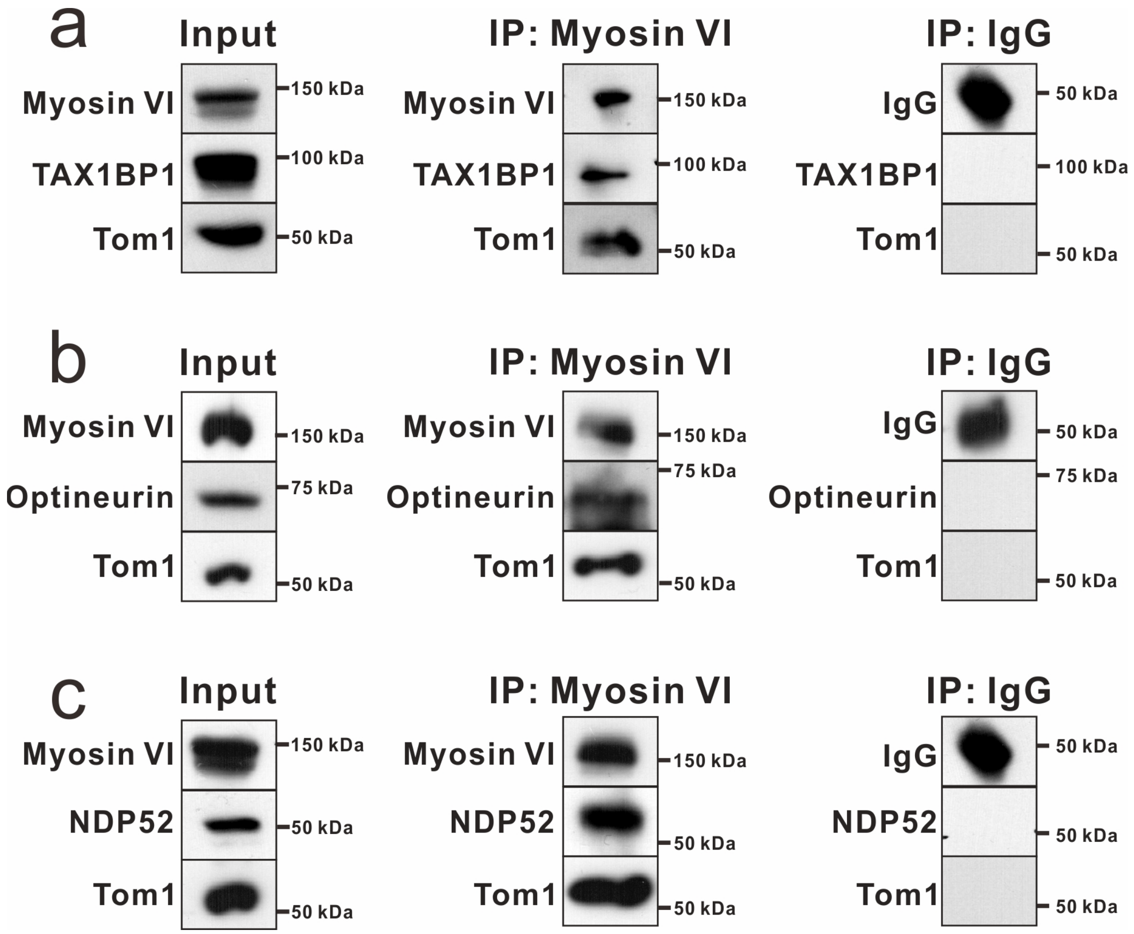

Supplementary Figure 10. Myosin VI can associate with Tom1 and autophagy receptors TAX1BP1, Optineurin and NDP52 forming ternary complexes at endogenous levels. (**a**-**c**) Co-immunoprecipitation assays showing that Myosin VI can interact with Tom1 and autophagy receptor TAX1BP1 (**a**), Optineurin (**b**), or NDP52 (**c**) at endogenous levels in vivo. In this assay, cell extracts were prepared from HEK293T cells, and 5% of each extracts were used as loading controls (left panel). Immunoprecipitation was performed under native conditions using an antibody to the tail region of Myosin VI (central panel), then run out on a 4-15% gradient gel, blotted, and analyzed using antibodies to Myosin VI, Tom1, TAX1BP1, NDP52 and Optineurin,

respectively. Control immunoglobulin G (IgG) immunoprecipitation (right panel) was performed using a normal mouse IgG. Source data are provided as a Source Data file.

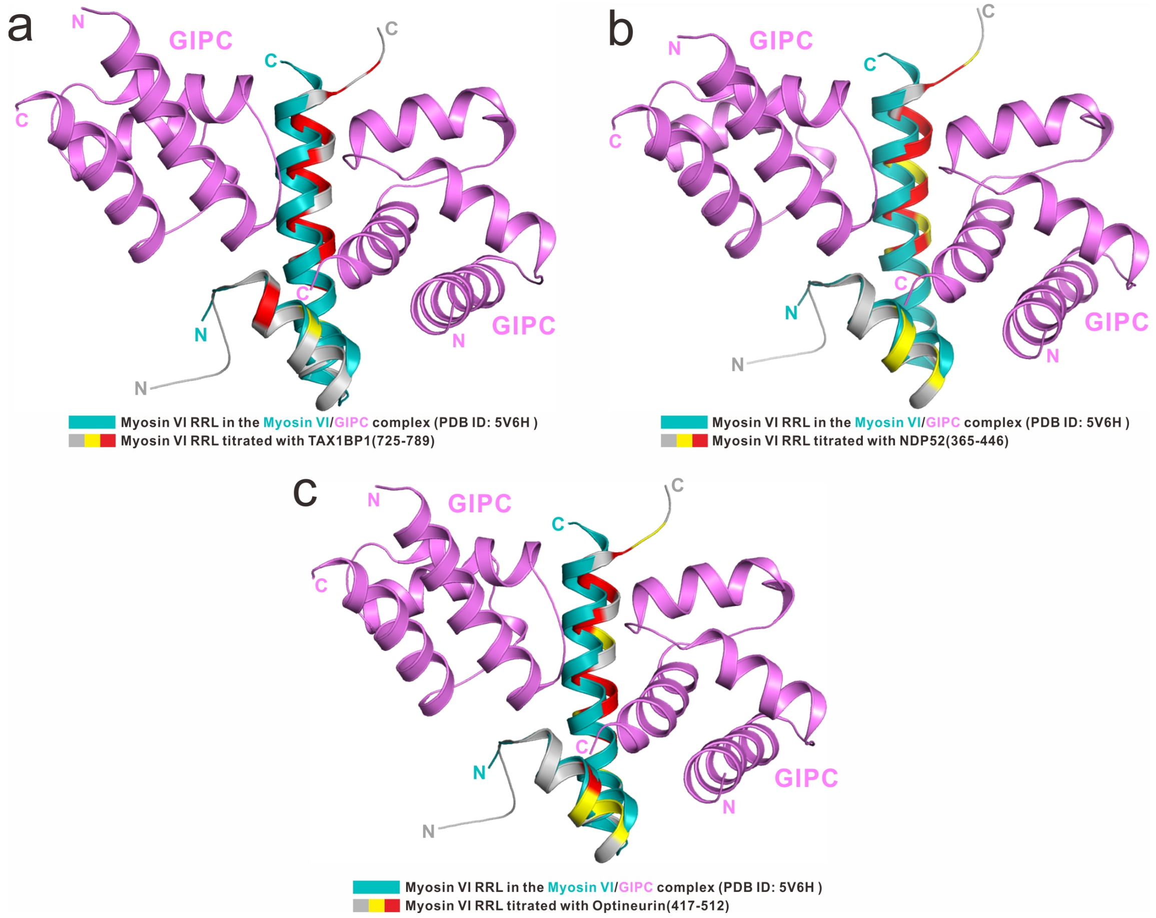

Supplementary Figure 11. The Myosin VI RRL motif uses an overlapped binding interface to interact with autophagy receptors and GIPC. (**a**) Ribbon diagram showing the comparison of the binding interface of Myosin VI RRL motif in interacting with TAX1BP1(725-789) revealed by NMR titration experiments in **Supplementary Fig. 9b** and that of Myosin VI RRL motif in binding to GIPC uncovered by the Myosin VI/GIPC complex structure (PDB ID: 5V6H). In this drawing, the Myosin VI RRL motif in the Myosin VI/GIPC complex (cyan) is aligned with the NMR structure of Myosin VI(1071-1122) (PDB ID: 2N10), which is colored based on different chemical shift

changes induced by TAX1BP1-binding in **Supplementary Fig. 9b**. (**b**) Ribbon diagram showing the comparison of the binding interface of Myosin VI RRL motif in interacting with NDP52(365-446) revealed by NMR titration experiments in **Supplementary Fig. 9d** and that of Myosin VI RRL motif in binding to GIPC uncovered by the Myosin VI/GIPC complex structure (PDB ID: 5V6H). (**c**) Ribbon diagram showing the comparison of the binding interface of Myosin VI RRL motif in interacting with Optineurin(417-512) revealed by NMR titration experiments in **Supplementary Fig. 9f** and that of Myosin VI RRL motif in binding to GIPC observed in the Myosin VI/GIPC complex structure (PDB ID: 5V6H).

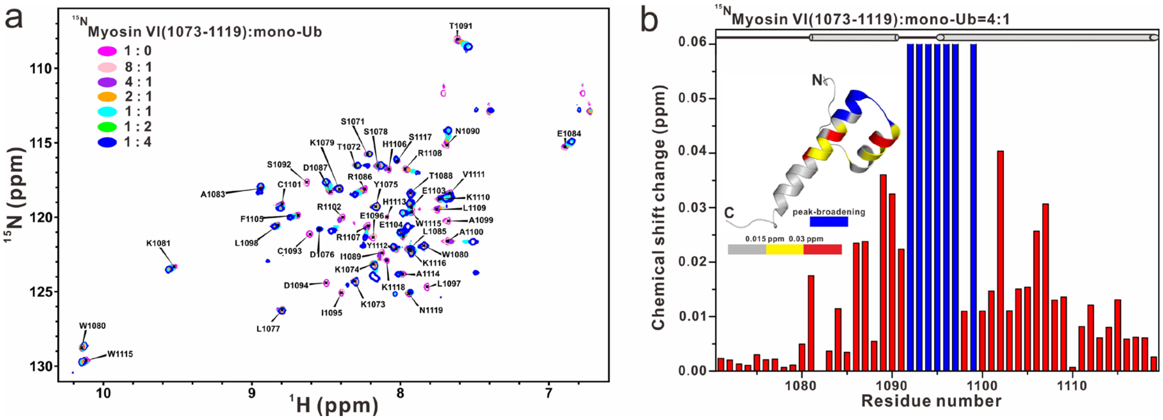

Supplementary Figure 12. NMR-based characterizations of the interaction between Myosin VI(1073-1119) and mono-ubiquitin. (**a**) Superposition plots of the ^{1}H-^{15}N HSQC spectra of Myosin VI(1073-1119) titrated with mono-ubiquitin at different molar ratios. (**b**) A plot of backbone amide chemical shift differences and peak-broadenings as a function of the residue number of Myosin VI(1073-1119) between the wild-type and the protein titrated with mono-ubiquitin at the molar ratio of 4:1. In this panel, the secondary structures and amino acid sequence of Myosin VI(1073-1119) are also indicated at the

top of the figure, and the insert shows the shift changes and peak-broadenings mapped onto a representative NMR structure of Myosin VI(1071-1122) (PDB ID: 2N10). In this representation, the residues with disappeared NMR peaks due to peak-broadenings are shown in blue, and the combined ^{1}H and ^{15}N chemical shift changes are defined as:

$$(3)\ \Delta_{ppm} = [(\Delta\delta_{HN})^2 + (\Delta\delta_N \times \alpha_N)^2]^{1/2}$$

Where $\Delta\delta_{HN}$ and $\Delta\delta_N$ represent chemical shift differences of amide proton and nitrogen chemical shifts of the each residue of Myosin VI(1073-1119). The scaling factor (α_N) used to normalize the ^{1}H and ^{15}N chemical shift is 0.17. Source data are provided as a Source Data file.

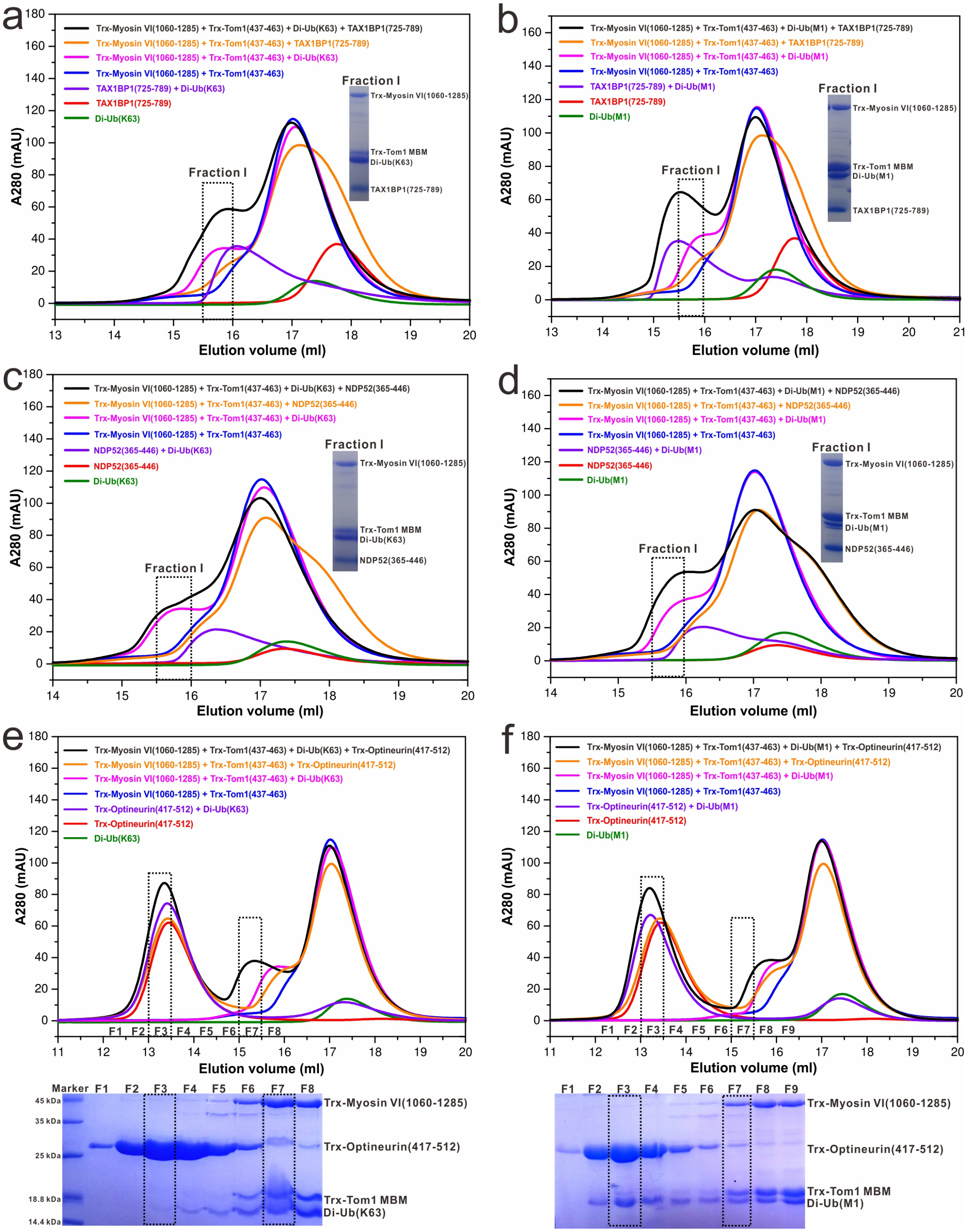

Supplementary Figure 13. K63-linked and M1-linked ubiquitin chains can promote the associations of autophagy receptors TAX1BP1, NDP52 and Optineurin with Myosin VI in vitro. (**a** and **b**) Analytic gel filtration chromatography coupled with SDS-

PAGE analysis using purified proteins, revealed that in the presence of K63-linked di-ubiquitin (**a**), or M1-linked di-ubiquitin (**b**), TAX1BP1(725-789) can readily associate with Myosin VI(1060-1285) and Tom1(437-463) to form quaternary complexes. In each panel, the insert shows the SDS-PAGE combined with Coomassie-blue staining analysis of the protein components of the indicated Fraction I fraction collected from the analytic gel filtration chromatography experiment of the Myosin VI(1060-1285)/Tom1(437-463)/di-ubiquitin/TAX1BP1(725-789) mixture (the black curve). Source data are provided as a Source Data file. (**c** and **d**) Analytic gel filtration chromatography coupled with SDS-PAGE analysis using purified proteins, revealed that in the presence of K63-linked di-ubiquitin (**c**), or M1-linked di-ubiquitin (**d**), NDP52(365-446) can readily associate with Myosin VI(1060-1285) and Tom1(437-463) to form quaternary complexes. In each panel, the insert shows the SDS-PAGE combined with Coomassie-blue staining analysis of the protein components of the indicated Fraction I fraction collected from the analytic gel filtration chromatography experiment of the Myosin VI(1060-1285)/Tom1(437-463)/di-ubiquitin/NDP52(365-446) mixture (the black curve). Source data are provided as a Source Data file. (**e** and **f**) Analytic gel filtration chromatography coupled with SDS-PAGE analysis using purified proteins, revealed that K63-linked di-ubiquitin (**e**), and M1-linked di-ubiquitin (**f**), can weakly promote the association of Optineurin(417-512) with Myosin VI(1060-1285). In each panel, the lower part shows the SDS-PAGE combined with Coomassie-blue staining analysis of the protein components of the indicated fractions collected from the analytic gel filtration chromatography experiment of the Myosin VI(1060-1285)/Tom1(437-463)/di-

ubiquitin/Optineurin(417-512) mixture (the black curve). Source data are provided as a Source Data file.

Supplementary Table 1. List of primer sequences used in this study.

Fragments	Primer sequences (5' to 3')
Tom1_1_BamH1_F	GTGGATCCATGGACTTTCTCCTG
Tom1_1_Hind3_F	GTAAGCTTATGGACTTTCTCCTG
Tom1_493_BamH1_R:	CGGGATCCTCATAAGGCAAACAGC
Tom1_493_Hind3_R	CGAAGCTTTCATAAGGCAAACAGC
Tom1_215_BamH1_F	CGGGATCCGAACAGATTGGGAAGCTGC
Tom1_392_BamH1_F	GTGGATCCACAGACGGCCTGGCTG
Tom1_392_Hind3_R	TCAAGCTTTCATGTTGCTTGGGGGGCT
Tom1_463_EcoR1_R	CGGAATTCTCAGCTGGAGAGGTTGGGC
Tom1_437_EcoR1_R	GCGAATTCTCACCCCTTAGGCTCTTCCG
Tom1_437_BamH1_F	GAGGATCCGGGGTCACCAGCGAAG
Tom1_464_BamH1_F	GAGGATCCCCCTCAGCTGAG
Tom1 (437-463)_L448E_F	CAAATTCGAGGAAGAACGGGC
Tom1 (437-463)_L448E_R	TCAAATTTACCTTCGCTGGTG
Tom1 (437-463)_R451E_R	GTCCGCGGCTTTGGCTTCTTCTTC
Tom1 (437-463)_R451E_F	CGATTGCCCAACCTCTCCAGC
Tom1 (437-463 deletion)_R	CTTAGGCTCTTCCGCATCATTACCC
Tom1 (437-463 deletion)_F	CCCTCAGCTGAGGGGCCCCCGGGTC
Myosin VI_1032_BamH1_F	GAGGATCCGCCCTGAGGATTGCCCAGAG
Myosin VI_1285_EcoR1_R	GTGAATTCCTACTTTAACAGACTCTG
Myosin VI_1285_Xho1_R	CGACTCGAGCTACTTTAACAGACTCTGCAGC
Myosin VI_1157_Hind3_R	CGAAGCTTTCAGGCAGGAATCTGAGCTGC
Myosin VI_1073_BamH1_F	TGGGATCCAAGAAATATGATCTTAGT
Myosin VI_1119_Hind3_R	GAAAGCTTCTAGTTCTTAGATTTCCAAG
Myosin VI_1060_Xhol1_F	CGCTCGAGCTGGTCCTGCTGTACTAGC
Myosin VI_1060_BamH1_F	ACGGATCCGGTCCTGCTGTACTAGCCA
Myosin VI(1157-1285)_W1193A_F	GTAAGAAAAAAGGCTGGGCGTATGC
Myosin VI(1157-1285)_W1193A_R	TCTGAGGGTCTTTGTACTGGTCGGC
Myosin VI(1157-1285)_E1207R_F	CGGCAAATGAGACTCCATCCTG
Myosin VI(1157-1285)_E1207R_R	GGCAATCCATGGTCCATCAAAA
