# Supplementary material for: Structure of Myosin VI/Tom1 complex reveals a cargo recognition mode of Myosin VI for tethering
Source: Nat Commun. 2019 Aug 1;10:3459. doi: 10.1038/s41467-019-11481-6 (PMC6673701; doi:10.1038/s41467-019-11481-6)
Supplement: Supplementary file 2 — Reporting Summary [file 41467_2019_11481_MOESM2_ESM.pdf]

nature research

Corresponding author(s): Lifeng Pan

Last updated by author(s): Jul 12, 2019

Reporting Summary

Nature Research wishes to improve the reproducibility of the work that we publish. This form provides structure for consistency and transparency in reporting. For further information on Nature Research policies, see Authors & Referees and the Editorial Policy Checklist.

Statistics

For all statistical analyses, confirm that the following items are present in the figure legend, table legend, main text, or Methods section.

n/a	Confirmed	
☐	☒	The exact sample size (*n*) for each experimental group/condition, given as a discrete number and unit of measurement
☐	☒	A statement on whether measurements were taken from distinct samples or whether the same sample was measured repeatedly
☐	☒	The statistical test(s) used AND whether they are one- or two-sided<br>*Only common tests should be described solely by name; describe more complex techniques in the Methods section.*
☒	☐	A description of all covariates tested
☒	☐	A description of any assumptions or corrections, such as tests of normality and adjustment for multiple comparisons
☐	☒	A full description of the statistical parameters including central tendency (e.g. means) or other basic estimates (e.g. regression coefficient) AND variation (e.g. standard deviation) or associated estimates of uncertainty (e.g. confidence intervals)
☒	☐	For null hypothesis testing, the test statistic (e.g. *F*, *t*, *r*) with confidence intervals, effect sizes, degrees of freedom and *P* value noted<br>*Give P values as exact values whenever suitable.*
☒	☐	For Bayesian analysis, information on the choice of priors and Markov chain Monte Carlo settings
☒	☐	For hierarchical and complex designs, identification of the appropriate level for tests and full reporting of outcomes
☒	☐	Estimates of effect sizes (e.g. Cohen's *d*, Pearson's *r*), indicating how they were calculated

Our web collection on statistics for biologists contains articles on many of the points above.

Software and code

Policy information about availability of computer code

Data collection	X-ray data processing was carried out using the HKL2000 or HKL3000 programs. Refinement of crystal structure was done using the phenix.refine program of Phenix. No custom software was used.
Data analysis	NMR data analysis was done using NMRpipe and Sparky. X-ray data analysis was done using CCP4, Phenix and COOT. Multi-angle light scattering data analysis was done using the ASTRA 6 software. Sedimentation velocity data was analyzed by SEDFIT. No custom software was used.

For manuscripts utilizing custom algorithms or software that are central to the research but not yet described in published literature, software must be made available to editors/reviewers. We strongly encourage code deposition in a community repository (e.g. GitHub). See the Nature Research guidelines for submitting code & software for further information.

Data

Policy information about availability of data

All manuscripts must include a data availability statement. This statement should provide the following information, where applicable:

- Accession codes, unique identifiers, or web links for publicly available datasets
- A list of figures that have associated raw data
- A description of any restrictions on data availability

We have included a data availability statement in the manuscript, and the coordinate and structure factor of the Myosin VI CBD/Tom1 MBM complex has been deposited in the Protein Data Bank with accession number 6J56.

Field-specific reporting

Please select the one below that is the best fit for your research. If you are not sure, read the appropriate sections before making your selection.

☒ Life sciences ☐ Behavioural & social sciences ☐ Ecological, evolutionary & environmental sciences

For a reference copy of the document with all sections, see nature.com/documents/nr-reporting-summary-flat.pdf

Life sciences study design

All studies must disclose on these points even when the disclosure is negative.

Sample size	No statistical method was used to determine the correct sample size. Instead the sample sizes were determined based on our experiences from previous studies using similar methodologies.
Data exclusions	No data was excluded from the study.
Replication	All biological experiments were carried out under clearly defined and standard conditions and were repeated at least twice whenever possible. All replication attempts were successful.
Randomization	The samples were randomly allocated.
Blinding	The in vitro experiments were not carried out blinded but most of them were done in parallel by at least two researchers.

Reporting for specific materials, systems and methods

We require information from authors about some types of materials, experimental systems and methods used in many studies. Here, indicate whether each material, system or method listed is relevant to your study. If you are not sure if a list item applies to your research, read the appropriate section before selecting a response.

Materials & experimental systems

n/a	Involved in the study	
☐	☒	Antibodies
☐	☒	Eukaryotic cell lines
☒	☐	Palaeontology
☒	☐	Animals and other organisms
☒	☐	Human research participants
☒	☐	Clinical data

Methods

n/a	Involved in the study	
☒	☐	ChIP-seq
☒	☐	Flow cytometry
☒	☐	MRI-based neuroimaging

Antibodies

Antibodies used	anti-mCherry, anti-GFP, anti-Flag, anti-Myosin VI, anti-Tom1, anti-TAX1BP1, anti-NDP52, anti-Optineurin, anti-IgG
Validation	Rabbit anti-mCherry(1:3000, Abcam, ab183628), Mouse anti-GFP(1:3000, Abmart, m20004-M/L), Mouse anti-Flag(1:3000, sigma, F1804), Mouse anti-Myosin VI (SANTA CRUZ, sc-393558, 1:100), Rabbit anti-Myosin VI (proteintech, 26778-1-AP, 1:1000), Rabbit anti-Tom1 (Abcam, ab99356, 1:1000), Rabbit anti-TAX1BP1 (Abcam, ab245636, 1:2000), Rabbit anti-NDP52 (Abcam, ab68588, 1:1000), Rabbit anti-Optineurin (Abcam, ab213556, 1:1000), Mouse anti-IgG (SANTA CRUZ, sc-2025, 1:100).

Eukaryotic cell lines

Policy information about cell lines

Cell line source(s)	HEK293T and HeLa cell lines were sourced from CBTCCAS.
Authentication	Authentication was not performed as none of the cells used have been listed in the commonly misidentified lines
Mycoplasma contamination	All cell lines were regularly tested for mycoplasma using the MycAwayTM-Color One-Step Mycoplasma Detection Kit (YEASEN M17371)
Commonly misidentified lines (See ICLAC register)	No misidentified lines were used in this study
